# Measuring the Interaction Between the Macro- and Micro-Vasculature

**DOI:** 10.3389/fcvm.2019.00169

**Published:** 2019-11-22

**Authors:** Rachel E. Climie, Antonio Gallo, Dean S. Picone, Nicole Di Lascio, Thomas T. van Sloten, Andrea Guala, Christopher C. Mayer, Bernhard Hametner, Rosa Maria Bruno

**Affiliations:** ^1^INSERM, U970, Paris Cardiovascular Research Center (PARCC), Paris Descartes University, Paris, France; ^2^Baker Heart and Diabetes Institute, Melbourne, VIC, Australia; ^3^Menzies Institute for Medical Research, University of Tasmanian, Hobart, TAS, Australia; ^4^Cardiovascular Prevention Unit, Department of Endocrinology and Metabolism, Pitié-Salpêtrière Hospital, Paris, France; ^5^Laboratoire d'imagerie Biomédicale, INSERM 1146 - CNRS 7371, Sorbonne University, Paris, France; ^6^Institute of Clinical Physiology, National Research Council, Pisa, Italy; ^7^Cardiovascular Research Institute Maastricht and Department of Internal Medicine, Maastricht University Medical Centre, Maastricht, Netherlands; ^8^Department of Cardiology, Hospital Universitari Vall d'Hebron, Vall d'Hebron Institute of Research, Barcelona, Spain; ^9^AIT Austrian Institute of Technology GmbH, Center for Health & Bioresources, Biomedical Systems, Vienna, Austria

**Keywords:** methods, microvascular, macrovascular, wave intensity analysis, brain, kidney, retina

## Abstract

Structural and functional dysfunction in both the macro- and microvasculature are a feature of essential hypertension. In a healthy cardiovascular system, the elastic properties of the large arteries ensure that pulsations in pressure and flow generated by cyclic left ventricular contraction are dampened, so that less pulsatile pressure and flow are delivered at the microvascular level. However, in response to aging, hypertension, and other disease states, arterial stiffening limits the buffering capacity of the elastic arteries, thus exposing the microvasculature to increased pulsatile stress. This is thought to be particularly pertinent to high flow/low resistance organs such as the brain and kidney, which may be sensitive to excess pressure and flow pulsatility, damaging capillary networks, and resulting in target organ damage. In this review, we describe the clinical relevance of the pulsatile interaction between the macro- and microvasculature and summarize current methods for measuring the transmission of pulsatility between the two sites.

## Introduction

High blood pressure (BP; hypertension), is the leading modifiable risk factor for the global burden of disease (1) and accounts for 9.4 million deaths worldwide each year (2), mostly due to cardiovascular disease (CVD) (3). Associated with raised BP is structural and functional dysfunction in both the macro- and microvasculature. In the macrovasculature this manifests as an increase in intima–media thickness (IMT) (4–7), accompanied by lumen enlargement (5–7) and increased stiffness in proximal elastic arteries (8) but not in distal muscular arteries (4–6). In the microvasculature, vasoconstriction, eutrophic remodeling (characterized by increased media-to-lumen ratio or wall-to-lumen ratio with no change in cross-sectional wall area) (9), alterations in distensibility, decreased vasodilatory reserve and rarefaction are evident in those with essential hypertension (10–12). Such changes in the vessels are likely to play a contributory role to hypertension-related organ damage and elevated CVD risk.

**Figure 1:**
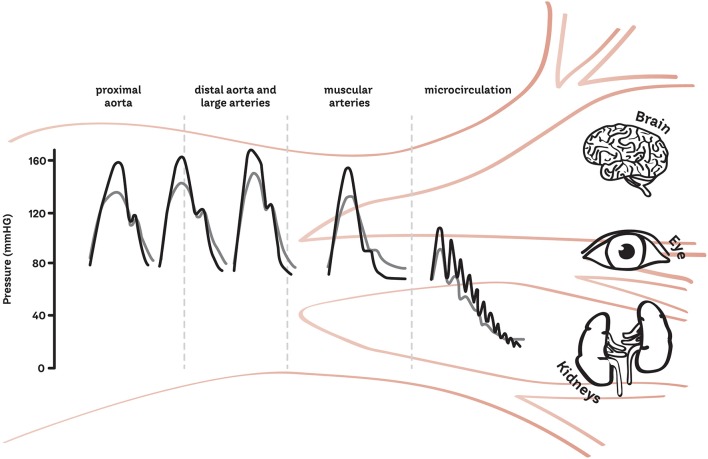
Schematic of the transmission of pulsatility from the macro to the micro-vasculature. The gray line represents the healthy vasculature and the black represents the increase in pressure and pulsatility which may occur with age or in disease states.

In a healthy cardiovascular system, the elastic properties of the large arteries ensure that pulsations in pressure and flow generated by cyclic left ventricular contraction are dampened, so that less pulsatile pressure and flow are delivered at the microvascular level. However, in response to aging (13, 14), hypertension and other disease states such as dyslipidemia and diabetes mellitus (15, 16), arterial stiffening limits the buffering capacity of the elastic arteries, thus exposing the microvasculature to increased pulsatile stress (17, 18). This is thought to be particularly pertinent to high flow/low resistance organs such as the brain and kidney, which may be sensitive to excess pressure and flow pulsatility, damaging capillary networks and resulting in target organ damage (19–24) (Figure 1). However, to our knowledge, few studies (25–27) have examined the macro- and micro-vasculature directly to determine whether there is transmission of pulsatility. This is an opportunity for future work as understanding the interaction between the macro- and microvasculature will provide targets for future treatment and management strategies aimed at limiting the pulsatility transmission to target organs, thus reducing target organ damage and ultimately improving clinical outcomes. In this review, we describe the clinical relevance of the pulsatile interaction between the macro- and microvasculature and summarize current methods for measuring the transmission of pulsatility between the two sites.

## Clinical Relevance of the Pulsatile Interaction Between the Macro- and Micro-Vasculature

The function of the aorta is to receive blood from the left ventricle and supply it to the systemic circulation. The proximal aorta achieves this by expanding during systole, which is made possible due to the highly elastic wall structure. The reservoir effect of the aorta allows a portion of the stroke volume ejected during systole to be temporarily stored and then propelled to the systemic circulation during diastole via recoil of the elastic arterial wall. Otherwise known as the Windkessel effect, this allows the aorta to provide continuous blood flow to the systemic circulation throughout the cardiac cycle and ensures the pulsatility of flow is reduced by the buffering effect of the reservoir (18). However, this reservoir function is highly dependent on (a) the stiffness and (b) the geometry of the arteries (28, 29), and is reduced in disease states.

- Arterial stiffness refers to the level of arterial compliance and vessel wall properties. A stiffer aorta will have a reduced reservoir capacity and a larger proportion of the ejected stroke volume will flow through the arterial system during systole, resulting in both intermittent pressure and flow as well as excessive pressure and flow pulsatility. This may contribute to target organ damage via remodeling, capillary rarefaction, and microvascular ischemia (30). The gold standard method to non-invasively quantify arterial stiffness is carotid-femoral pulse wave velocity (cfPWV). cfPWV is the quantification of time delay between carotid and femoral waveforms, divided by the distance covered. Other methods for measuring PWV in the large arteries exist including cuff-based techniques and phase-contrast magnetic resonance imaging (MRI). Moreover, other parameters, such as aortic strain and distensibility may provide an alternative description of large artery stiffness (31).
- The enlargement of the large arteries (i.e., thoracic aorta and common carotid artery) with aging and hypertension is generally due to the fracture of the load-bearing elastin fibers due to the fatiguing effect of both the steady and pulsatile tensile stress. Vascular smooth muscle cell (VSMC) growth and apoptosis may also be involved, as the cyclic, pulsatile strain on the vessels is also a determinant of gene expression and growth of VSMCs *in vitro* (32, 33). The enlargement of large proximal arteries is suggested to be a compensating mechanism, ensuring that a certain level of arterial compliance is maintained (29, 34, 35). However, when excessive (aneurysm), it may lead to major adverse aortic events such as dissection and rupture (36). Interestingly, the effect of pulsatile mechanical load on arterial remodeling has been observed in large elastic arteries but not in more distal, muscular arteries (radial). Large artery dimension and shape can be quantified non-invasively by MRI and ultrasound.

### The Brain

Recent work suggests that aortic stiffness and pulsatile hemodynamics are related to cerebral small vessel disease development (30, 37–41). Cerebral small vessel disease is a range of neuroimaging findings (including white matter hyperintensities and lacunes of presumed vascular origin, cerebral microbleeds, perivascular spaces, and total cerebral atrophy) thought to arise from disease affecting the perforating cerebral arterioles, capillaries and venules, and the resulting brain damage in the cerebral white and deep gray matter (42). In the Age, Gene/Environment Susceptibility (AGES)—Reykjavik study, higher aortic stiffness was associated with an increase in flow pulsatility transmission to the cerebrovascular circulation (30). In middle-aged and older adults, aortic stiffness and pressure pulsatility were associated with progression of neurovascular disease and cognitive decline (43). The association between mean blood flow and its pulsatility and mild cognitive impairment was also reported in a cross-sectional study (44) based on 4D flow MRI, the reference technique for flow evaluation especially in complex vascular territories, such as inside the skull. Additionally, excess pressure, analogous to left ventricular flow, was related to gray matter atrophy in healthy subjects (45).

### The Kidney

The relationship between arterial stiffness and pulsatility in the kidneys has been demonstrated in several observational studies [summarized in (46)]. These studies evaluated the association between arterial stiffness and chronic kidney disease progression, with conflicting results in those with type 2 diabetes (T2D) (47, 48), hypertension (49), elderly (50), healthy middle-aged (51, 52), and young adults (53). Interestingly, in both middle age and elderly subjects, an increase in brachial pulse pressure was associated with accelerated renal function decline (50, 52) and in patients with T2D, excess pressure was related to exercise-induced albuminuria (24). However, the most convincing evidence on the clinical relevance of the macro-microvascular interaction for kidney function comes from a cross-sectional analysis of the AGES study cohort (54). In 367 older adults aged 72–92 years, a mediation analysis demonstrated that 34% of the relationship between aortic stiffness and estimated glomerular filtration rate (eGFR) was mediated by increased pulsatility index in the renal artery, assessed via MRI flow waveform measurements. Aortic stiffness was found to induce kidney damage mostly by means of an increased flow pulsatility transmission (54). Interestingly, high pulsatility mediates PWV-induced eGFR decline but the effect on microalbuminuria accrual is less clear. Thus, it is conceivable that the deleterious macro- microvascular interaction in diseases such as T2D may be responsible for the increasingly higher prevalence of normoalbuminuric/eGFR decline, an emerging phenotype in contemporary epidemiology of diabetic nephropathy (55). However, this hypothesis needs to be tested in future studies.

### The Retina

The retina is a unique site where the microcirculation can be imaged directly, providing an opportunity to study *in vivo* the structure and pathology of the human circulation. The retina is characterized by a dual blood supply: the inner layers are supplied by the retinal arteries derived from the central retinal artery; the outer retina, being avascular, depends on choroidal circulation (56). These two vascular systems being completely independent, present specific anatomical and physiological characteristics, resulting in higher perfusion rate in the choroidal vasculature and higher resistance at the inner retinal level (57). As a consequence, the outer retinal layers may be more exposed, and damaged by increased flow pulsatility related to increased large artery stiffness, although this hypothesis needs to be confirmed. Large artery stiffness has been shown to be related to diabetic retinopathy (58), age-related macular degeneration (59) and retinal microvascular impairment (60, 61). Exaggerated pulsed retinal capillary flow, in contrast to unchanged mean retinal capillary flow, and stiffer wall properties of retinal arterioles has been observed in patients with treated resistant hypertension compared with patients with grade 1–2 hypertension (62). Furthermore, retinal PWV discriminated between patients with mild hypertension and those with normal or high normal BP (63, 64) and may be related to large artery PWV.

## The Macrovasculature and Pulsatile Hemodynamics

With advancing age, there is gradual degradation and fracture of the elastin fibers in the arterial wall, leading to dilation, and stiffening of large elastic arteries (aorta, carotid). In a study of aortic sections from a range of animal species, a higher number of cardiac cycles across the lifespan (heart rate x age) were associated with greater disorganization of elastin, demonstrating how the stress of each heart beat gradually alters arterial wall structure causing loss of aortic buffering function (65), in a process often assimilated to material fatigue due to cyclic stress. Thus, aortic stiffness seems to precede, and induce, pulse pressure elevation and hypertension (66–69). In parallel, sustained increases in BP lead to changes to smooth muscle cell organization and the extra-cellular matrix, resulting in greater arterial stiffness (70, 71).

The relation between vessel geometry and distensibility and local pulse pressure is highly debated. In a multivariable analysis of a cohort of normotensive, and treatment-naïve hypertensive patients, common carotid artery diameter and carotid IMT were positively related to carotid pulse pressure, as well as heart rate and age (7). Accordingly, a cross-sectional MRI study of 100 apparently healthy adults showed aortic dilation, elongation, and reduced curvature in older age. Each of the geometric changes were strongly related to higher systolic BP (72, 73). In contrast other data, such as the 16-year follow up from the Framingham Heart Study and the 20-year follow-up from the Healthy Coronary Artery Risk Development in Young Adults study (74, 75), support the notion that higher central aortic pulse pressure is associated with lower aortic diameter (76–78). Finally, an MRI study in young-middle aged adults with isolated systolic hypertension (and thus elevated pulse pressure), suggested that it is rather the mismatch between aortic stiffness and diameter, which could explain elevated pulsatility (77).

An emerging determinant of increased transmission of pressure and flow pulsatility at the microvasculature level occurring with age and risk factors is the reduced impedance mismatch between large and medium-sized muscular arteries. The impedance is the relationship between pressure and flow. In the context of large arteries, the characteristic impedance is often used to quantify the amount of reflection generated from the passage of a wave. At a location where characteristic impedance changes, often called impedance mismatch, a reflected wave is generated. Larger and more elastic vessels have lower characteristic impedance. According to the so-called stiffness gradient hypothesis, in healthy young individuals, when aortic stiffness is lower than that of medium-sized muscular conduit arteries, some suggest that partial pressure wave reflections are generated at the transition of these segments, resulting in attenuated pulse pressure transmission and possible protection of microcirculation (34, 79). By increasing large but not small artery stiffness, aging and risk factors limit or even reverse this gradient, attenuating distal reflection and thus increasing the amount of forward pressure wave transmitted to the microcirculation, potentially leading to increased organ damage. This hypothesis was supported by a prospective study in dialysis patients and demonstrated that a reduced stiffness gradient is associated with increased cardiovascular events (80). Furthermore, a reduced stiffness gradient was observed in patients with T2D (81). However, others have shown that aortic-brachial stiffness gradient had little or no impact on wave reflection (evaluated as augmentation index) and left ventricular hypertrophy (82).

## The Microvasculature and Pulsatile Hemodynamics

The microcirculation has long been thought to only be representative of peripheral vascular resistance (i.e., steady state, as expressed as the ratio between mean arterial pressure and cardiac output). However, the pulsatile component of the BP curve (i.e., pulse pressure) influences the entire arterial tree, including small arteries. Vasoconstriction of the arterioles may increase the amplitude of wave reflection, resulting in an increase in central (aortic) pulse pressure. However, an alternate explanation for an increase in central pulse pressure may be an increase in the forward compression wave (83–85). Conversely, endothelial cells, and pericytes in the microvasculature may respond to increased pulsatile flow by compensatory mechanisms, such as increased production of nitric oxide and activation of cyclooxygenase-2, which are concomitant with endothelin-1 and prostacyclin decrease (86). When nitric oxide availability in the microcirculation is reduced in conditions such as increased oxidative stress (as in aging and hypertension) or hyperglycemia (as in T2D), the impact of large artery flow pulsatility in the microcirculation may be greater (87). The microcirculation also represents the very early site of expression of CVD, by means of a chronic inflammation state. The overexpression of reactive oxygen species leads to an increased myogenic tone and is responsible for microvascular remodeling in hypertension (88). This inflammatory state may be also modulated by peculiar flow conditions, such as an atheroprotective flow that was shown to induce miRNAs, which are involved in the downregulation of pro-inflammatory and upregulation of anti-inflammatory molecules (89).

## Cross Talk Between the Macro- and Micro-Vasculature and Methods to Measure the Interaction

**Figure 2:**
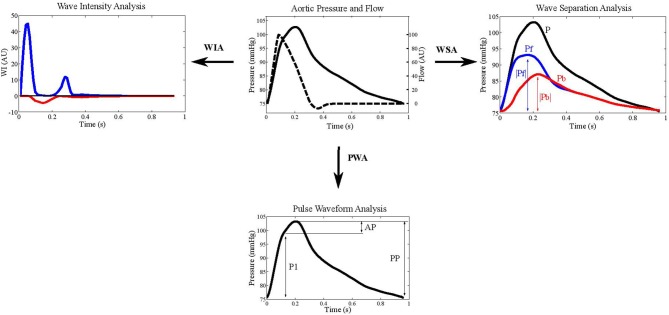
Example aortic pressure and flow waveforms depicted in wave intensity analysis (WIA), wave separation analysis (WSA), and pulse wave analysis (PWA). The blue lines indicate forward pressure (Pf) and the red lines represent backward pressure (Pb). Augmentation index is calculated as augmented pressure (AP) divided by pulse pressure (PP).

To investigate the interaction between the macro- and microvasculature, knowledge of the fluid dynamics between these regions in the human body is essential. Following the wave transmission approach, arterial pressure, and flow are the result of superimposing forward and backward traveling waves. Thus, it is desirable to quantify waves traveling in the forward direction from large to small arteries, as well as to quantify reflected waves traveling from the microcirculation back into larger arteries (Figure 2).

Augmentation index (AIx), defined as the difference between the shoulder on the pressure wave and systolic pressure divided by pulse pressure, has been widely used as a measure of wave reflections (29) (Figure 2). An advantage of AIx is its non-dimensionality, requiring neither calibration of BP nor measurement of blood flow velocity. BP waveforms can be obtained using non-invasive tonometry at the location of the carotid- or radial arteries, or by oscillometric BP recordings at the brachial level (90); however the validity of AIx as a measure of reflection is uncertain as it is also influenced by PWV and other factors. It has been suggested that AIx may be more indicative of arterial compliance and reservoir function than wave reflection (91). Indeed, in healthy individuals, no relationship between AIx and the “gold standard” measures of wave reflection calculated from pressure and flow data were found (85). Furthermore, using a computational model of the circulation, it was recently demonstrated that myocardial shortening velocity and large artery stiffness are the main determinants of AIx (92). Thus, despite AIx being used extensively in cardiovascular research and its predictive value for cardiovascular outcomes (93), the available evidence suggests that AIx may not suitably represent the interaction between macro-and microvasculature and supports the use of wave separation and intensity techniques (94, 95). Following the wave transmission approach, methods for the separation of pressure, and flow waveforms into their forward and backward components have been presented, and indices for the quantification of meaningful descriptors have been developed (96).

### Wave Separation Analysis

Westerhof et al. introduced the impedance method for wave separation analysis (28) (Figure 2). Assuming a stable cardiovascular condition, the characteristic impedance Z_c_ is estimated in the frequency domain as high frequency limit of the input impedance. Subsequently, forward (P_f_) and backward (P_b_) traveling pressure can be expressed, based on measured pressure (P) and flow (Q), as:

$$\begin{array}{l} {\text{P}}_{\text{f}}=\left(\text{P}+{\text{Z}}_{\text{c}}^{*}\text{Q}\right)/2\\ {\text{P}}_{\text{b}}=\left(\text{P}-{\text{Z}}_{\text{c}}^{*}\text{Q}\right)/2 \end{array}$$

where P is pressure and Q is volume flow.

Alternatively, wave separation can also be performed in the time domain. In this case, wave speed instead of wave characteristic impedance is required. Usually, the amplitudes of P_f_ and P_b_ or their ratio P_b_/P_f_, also denoted as reflection magnitude, are used as indices for the quantification of the pressure waves (97). Reflection magnitude showed a strong predictive value both for cardiovascular events and new-onset heart failure in a large community sample (98). In particular, P_f_ amplitude has been associated with increased cardiovascular event incidence, beyond traditional risk factors and arterial stiffness (99).

### Wave Intensity Analysis

Wave intensity analysis (WIA) is increasingly employed in the study of the cardiovascular system, providing additional, and complementary information to the standard vascular evaluation (Figure 2). Wave intensity represents the instantaneous power carried by the pulse wave per unit cross sectional area traveling from the heart to the periphery. The energy associated with this wave is the result of the kinetic energy related to the blood flow and the potential energy linked to the expansion of the arterial wall (97).

The WIA implementation requires the acquisition of the pressure and the flow velocity waveforms at a specific arterial site. The wave intensity signal is then obtained by multiplying the time derivative of pressure by the time derivative of blood velocity (100). As a consequence, absolute wave intensity values can characterize the traveling waves in terms of direction, discriminating between forward waves originating from the heart and backward ones arising from reflections sites. Furthermore, since different changes in pressure and flow velocity lead to the compression or the expansion of the vessel, both the forward and the backward fronts can be characterized in terms of compression and expansion waves (101).

The WIA signal in the aorta (101) presents a first positive and prevailing peak in the early systolic phase, caused by the simultaneous increasing of pressure and flow velocity originating from left ventricle ejection (102). This local maximum is followed by a small negative peak, generated by concomitant increase in pressure and decrease in blood flow and is representative of the backward compression wave originating from the reflection of the forward compression wave from more distal points (103). Finally, at the end-systolic phase, the wave intensity signal shows a second positive peak, smaller than the first one, and caused by the simultaneous decrease in pressure and flow velocity (forward expansion wave) (102).

The analysis of the wave intensity signal provides quantitative information about the energy transfer along the arterial tree; therefore, this approach may be useful for obtaining information about the interaction between macro- and micro-vasculature (104). Currently, most literature concerns the cerebral circulation. WIA was used to assess changes in the cerebral vasomotor tone as a consequence of a hypercapnia status, which is known to alter cerebral resistance. In this study, the amplitude of the negative peak, both considering it as an absolute value or divided by the amplitude of the first positive peak (reflection index), was significantly decreased following increase carbon dioxide concentration, indicating an association between reduction in reflections and cerebral vasodilation (105). This result is in line with other work focused on the effects of two different hypertensive treatments. WIA was employed at the carotid artery level and the WIA-derived reflection index was significantly lower for the treatment with a greater vasodilator action, as a consequence of an improved impedance matching in correspondence of bifurcations (106). In treated hypertensive patients, WIA-derived reflection index, but not reflection magnitude and AIx, predicted cardiovascular events independently of traditional risk factors (107). Furthermore, a recently published longitudinal study showed that the amplitude of the forward traveling wave, as assessed in mid- to late-life at the carotid artery level, predicts faster cognitive decline, independent from other cardiovascular risk factors (108).

Despite this evidence, some technical, and practical issues should be considered. Since invasive assessment of pressure and flow velocity waveforms is not feasible for widespread use (101, 109), non-invasive approaches have been proposed, using applanation tonometry to obtain the pressure curve and ultrasound pulsed wave Doppler imaging for the acquisition of the flow velocity (104, 110, 111). Alternatively, WIA can be implemented using diameter values instead of pressure following the mathematical theory reported in (112). This method has been applied at both the carotid and femoral artery (113) and represents a valid approach even in preclinical settings involving murine models, in which both the invasive and the standard non-invasive methods are more difficult to implement (114).

### Wave Power Analysis

A drawback of the wave intensity is that it is not a conserved quantity, i.e., it is sensitive to variations in the vessel diameter, leading to difficulties in analyzing wave transmission in the arterial tree. To overcome this problem, Mynard and Smolich proposed the wave power analysis as an alternative (115). To calculate wave power, volume flow instead of flow velocity is used. As for the other methods, forward and backward components of wave power can be derived to investigate wave transmission phenomena. Recently, wave power analysis was used to identify a higher aorto-carotid wave transmission in patients with reduced aortic distensibility after coarctation repair. This is of importance, as it is known that these subjects have an increased risk of cerebrovascular disease and stroke even after successful surgical treatment (116, 117). Extensive clinical validation is needed to understand the role of wave power analysis in the panorama of the other techniques assessing wave reflection.

## Methods for Measuring Pulsatility in the Microvasculature

Methods for measuring pulsatility in the macrovasculature are displayed in Table 1. Different methods are available to assess the microvascular pulsatile hemodynamics in low-resistance, high flow organs such as the brain (and retina) and the kidneys (Table 2).

**Table 1:** Methods used to determine pressure and flow pulsatility in the macrovasculature.

**Method**	**Description**	**Variables**	**Advantages**	**Disadvantages**
MRI	High resolution imaging	Arterial structure, blood flow velocity	Very high-resolution	Costly equipment, can only be used in specialist research or hospital settings
Ultrasound, high resolution echotracking methods	Single micrometer resolution during continuous measurements	Arterial structure, pulsatility index	Mobile equipment available	Costly equipment
Doppler ultrasonography	Employs Doppler effect to image movement of blood and velocity	Blood flow velocity	Mobile equipment available	Costly equipment, can only be used in specialist research or hospital settings
Applanation tonometry, pulse wave velocity, and analysis	Pressure sensor placed on palpable artery to record arterial waveform Proprietary algorithms used to derive central BP parameters	Arterial stiffness, central pulse pressure, augmented pressure, augmentation index	Central PP and wave parameters may give more useful clinical information that peripheral measurements	User dependent, results are dependent on pressure wave calibration method and device (algorithm)
Standard cuff BP	BP cuff placed around the upper arm or wrist Automated or manual measurement	Brachial PP (including possibility to measure variables over 24 h)	Clinically relevant, easy to measure	Central instead of brachial pulse pressure may be more clinically relevant
Oscillometric central BP	BP cuff placed around the upper arm, algorithms used to determine central BP Suprasystolic methods also available	Central pulse pressure, augmented pressure, augmentation index (including possibility to measure variables over 24 h)	Central pulse pressure and wave parameters may give more useful clinical information that peripheral measurements	Can be highly dependent on brachial BP measurement, results are dependent on pressure wave calibration method and device (algorithm)
Intra-arterial (invasive) catheter methods	Recordings taken during invasive hospital procedures, most commonly coronary angiography, or coronary artery bypass grafting	BP and Doppler flow velocity (if specialist pressure-flow wires are used)	High-quality invasive recordings	Difficult and expensive to collect the data, only suitable in specific patient populations

*BP, blood pressure; MRI, magnetic resonance imaging*.

**Table 2:** Methods used to determine pressure and flow pulsatility in the microvasculature.

**Method**	**Description**	**Variables**	**Advantages**	**Disadvantages**
**Brain**				
Cerebral vasoreactivity	Vasodilatory ability of the cerebral (micro)vasculature	Mean increase in blood flow or blood flow velocity after stimulation with either acetazolamide or CO_2_	Functional imaging; also possible at the level of the microvasculature with 7 Tesla MRI	Most methods available measure vasoreactivity at the level of large intracranial arteries, and not directly at the level of the microvasculature
Cerebral blood flow pulsatility	Blood flow pulsatility	Pulsatility index	Functional imaging; also possible at the level of the microvasculature with phase-contrast 7 Tesla MRI	Most methods available measure vasoreactivity at the level of large intracranial arteries, and not directly at the level of the microvasculature
Cerebral microvascular perfusion	Intravoxel incoherent motion MRI, a diffusion-weighted MRI technique without the use of contrast agents	Perfusion fraction, a measure for blood perfusion volume; and blood flow These variables are potentially sensitive to microvascular pathology	High signal-to-noise ratio and high spatial resolution; simultaneous assessment of tissue microstructure and microvasculature	Experimental tool
**Kidney**				
MRI	High resolution imaging	Arterial structure and blood flow velocity, vascular resistance, pulsatility index	Very high-resolution	Costly equipment, can only be used in specialist research or hospital settings
Renal Doppler sonography	Employs Doppler effect to analyze renal blood flow velocity pattern	Resistive index, pulsatility index, compliance index, renal acceleration time	Non-invasive technique; cost-effectiveness	Highly operator-dependent
Transesophageal Doppler	Employs Doppler effect to analyze renal blood flow velocity pattern	Resistive index, pulsatility index	Real-time measurement	Invasive procedure, specific training is needed
**Retina**				
Fluorescein angiography and indocyanine green angiography	Calculates the time of transition of a dye molecule throughout a microvascular segment	Vessel diameter (photo/video/mean transit time two-point fluorophotometry), mean transit time, arteriovenous passage	Coupled with Scanner laser ophthalmoscopy allows the direct measurement of retinal blood flow	Reliable data only if the vascular segment, diameter and volume of distribution satisfy specific conditions
Laser Doppler velocimetry	Measure of the maximum blood cell velocity in retinal vessels through the analysis of Doppler shifts	Blood velocity Estimated volumetric flow (based on the diameter of vessels >50 μm)	Useful to document physiologic changes in retinal perfusion	Very complex technique with multiple controls to manipulate, which makes it available only in research settings
Laser Doppler flowmetry	Using spectral analysis and wavelet transform	Blood flow velocity, pulsatility	Blood flow measurement is derived from red blood cells velocity and volume instead of diameter, thereby minimizing the variability due to different imaging methods for diameter calculation	Individual anatomy and local hematocrit may alter the blood flow estimation, a comparison between healthy and pathologic retina may be difficult
Scanning laser Doppler flowmetry	Integration of spectral analysis and red blood cell flow	Arterial structure, blood flow velocity	Non-invasive, *in-vivo*, both morphological and functional analysis	Mixed signal of retinal and choroidal tissue, limiting the interpretation of results, only available in research settings
Laser speckle flowgraphy	Measure of the blood flow based on the laser speckle phenomenon and mean blur rate pulse waveform analysis	Blood flow velocity, blowout time, blowout score	Quantitative ocular blood flow measurement *in vivo*	Arbitrary units implying difficult comparison with other techniques, subject compliance (good fixation) to obtain good images
Doppler optical computed tomography	Motion-contrast imaging based on backscattered light from retinal tissue High-resolution cross-sectional imaging	Arterial structure and anatomy, blood flow extracted from Doppler shift	Contactless and dye-free	Costly equipment, can only be used in specialist research or hospital setting, cannot be applied for *in vivo* real-time measurements, motion-sample dependent
Color Doppler	Quantification of blood velocities through Doppler effect	Resistive index, blood flow velocity	Ocular blood flow and blood velocity easily uncoupled	An increase in intraocular pressure may occur when the probe is applied on the closed eye, poor reproducibility

*MRI, magnetic resonance imaging*.

### The Brain

Most of the measures of pulsatility in intracranial arteries are based on MRI or transcranial doppler ultrasound.

### Cerebral Vasoreactivity

Cerebral vasoreactivity is a measure for the vasodilatory ability of the cerebral (micro)vasculature and is defined as the mean increase in blood flow or velocity after stimulation with either acetazolamide or CO_2_ (118). Cerebral vasoreactivity can be measured at the tissue level using blood oxygenation level dependent MRI, arterial spin labeling, or positron emission tomography (119, 120). In addition, cerebral vasoreactivity can be determined at the level of the large intracranial arteries via transcranial doppler ultrasound or phase contrast MRI (121) or in the small cerebral perforating arteries, using phase-contrast high resolution (7 Tesla) MRI (122).

### Cerebral Blood Flow Pulsatility

Cerebral blood flow pulsatility can be measured at the level of the carotid artery via MRI and ultrasonography. High carotid artery blood flow pulsatility is associated with MRI features of cerebral small vessel disease (e.g., lacunes) and worse cognitive performance (30, 123). In large cerebral arteries, in cerebral perforating arteries and arterioles, flow pulsatility can be assessed by phase-contrast MRI. In this region, characterized by complex arterial network, 4D-flow MRI sequences (44), by measuring blood velocity in three orthogonal directions and in large volume, may be superior to standard (1D) PC-MRI. Indeed, since they do not require a specific measurement location or velocity encoding direction, 4D-flow MRI is free of angle-dependent errors (velocity errors ensuing from the misalignment between velocity encoding and blood velocity). Another key result of encoding in three directions is the possibility to quantify complex flow patterns, which are related to local dilation (31, 35, 124, 125) and arterial wall disruption (126).

### Cerebral Microvascular Perfusion

Intravoxel incoherent motion MRI, a diffusion-weighted MRI technique without the use of contrast agents, can be used to assess cerebral microvascular perfusion (127). This technique enables assessment of both the parenchyma and microvasculature and is based on the diffusion of water molecules in parenchyma and incoherent motion of water molecules in the microvasculature (127). Intravoxel incoherent motion MRI has been used mainly to investigate the brain, but may also be used in other parts of the body (128). Although it was introduced in the mid-eighties (129) it is still experimental, but it can provide a high signal-to-noise ratio and high spatial resolution (127, 128). An advantage of this technique is the simultaneous assessment of tissue microstructure and microvasculature, and, therefore, of the interplay between brain tissue and vessels (130).

Higher cerebral pulsatility index has been shown to be associated with MRI features of cerebral small vessel disease (131) and cognitive impairment (132). Furthermore, a recent study using intravoxel incoherent motion MRI found that the microvascular properties of the hippocampus are altered in individuals with T2D (130), which may be related to worse cognitive function. While these biomarkers show promise for identifying individuals at elevated risk, their prognostic value needs to be confirmed in larger prospective studies. Cerebral vasoreactivity of small arteries/arterioles using 7 Tesla provides a direct functional measurement of the cerebral microvasculature and may be preferable for investigating the interaction between the macro- and microvasculature, but this technology is available only in few, specialized centers, and only proof-of concept studies have been performed.

### The Kidney

Renal hemodynamics are classically assessed by renal plasma flow, which is an invasive and time-consuming technique, including radiotracer intravenous administration (133, 134). More recently, non-invasive techniques, including ultrasound and MRI have been successfully applied (135) allowing a direct quantification of renal microvascular blood flow, together with structural characterization.

### Magnetic Resonance Imaging

Without the use of radiation, MRI allows for blood flow and velocity assessment via phase-contrast sequences and it can provide detailed 3D angiography. As such, MRI can quantify vascular resistance measures and pulsatility index (136). Moreover, blood oxygen level dependent MRI sequences allow for the measurement of kidney tissue hypoxia.

### Renal Doppler Sonography

**Figure 3:**
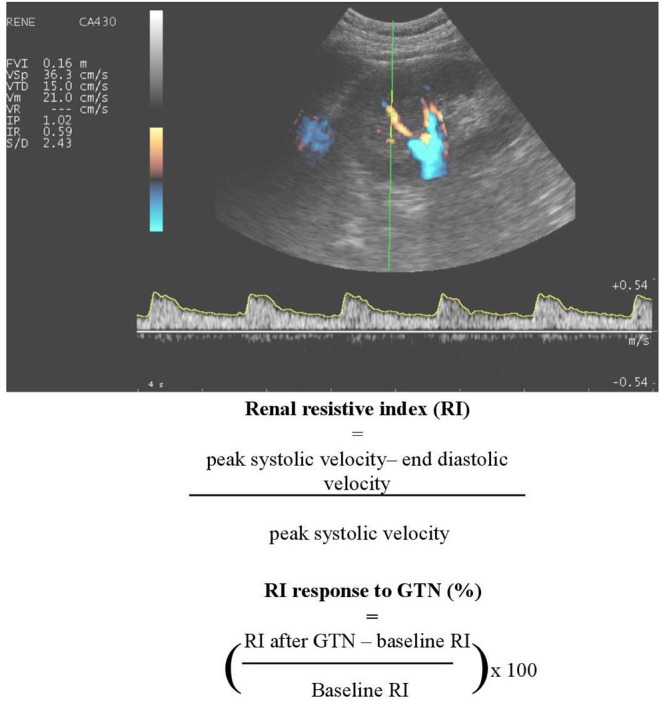
An example of renal Doppler sonography. A number of variables expressing flow pulsatility and vascular resistance can be determined, including renal resistive index (RI) and dynamic RI.

The most widely used technique for blood velocity assessment is Doppler ultrasound due to wide availability, non-invasive, and relatively easy use. Duplex ultrasound on the interlobar renal arteries allows for the measurement of a number of variables expressing flow pulsatility and vascular resistance, among which the most widely used is renal resistive index—RI—an angle-independent, semiquantitative parameter defined as [peak systolic velocity (PSV)-end diastolic velocity(EDV)]/PSV. The clinical significance of RI is still a matter of debate, since it may be determined by systemic hemodynamics, arterial compliance, PWV (137–139) or local flow pulsatility, rather than renal vascular resistance (140). However, this observation, which is usually seen as a limitation of the technique, might indeed make RI a good candidate to represent the interaction between the macro- and microvasculature, or rather its integrated effect on the kidney. Finally, RI is able to track drug-induced changes in renal hemodynamics (141). This led to the calculation of a dynamic RI, estimating renal vasodilatory capacity before and 5 min after nitrate-induced vasodilation (Figure 3).

### Transesophageal Doppler

A reduced systemic pulsatile blood flow is considered to hamper renal perfusion leading to acute kidney failure. Transesophageal Doppler allows the measurement of angle-dependent blood flow velocities (PSV, EDV, and mean diastolic velocity) and angle -independent indices (RI and pulsatile index) in the renal artery. Despite being an invasive procedure, the measurement can be done in real-time and images can be obtained in <5 min by trained personnel [summarized in (142)].

To date, a number of studies have demonstrated the prognostic role of RI, especially in T2D (143) and chronic kidney disease (144), whereas dynamic RI is associated with PWV and predicts microalbuminuria development in patients with hypertension and T2D (138, 145). Thus, at present, these measures may be useful renal biomarkers to investigate the interaction between the macro- and microvasculature. To our knowledge, the relationship between markers of renal pulsatility obtained using MRI and clinical outcomes has never been assessed, though this technique is promising and likely more accurate and reproducible than ultrasound-based ones.

### The Retina

Most widely used retinal microvascular variables include the central retinal arteriolar/venular diameters or equivalents (146), although more recent techniques allow a near-histological evaluation of the arteriolar wall (9). Recently, other measures of the retinal microvascular network geometry have been studied, e.g., tortuosity, bifurcation angles and optimality, and fractal dimensions (146), which are associated with diabetic retinopathy, stroke, and cognitive impairment (147). It is also possible to dynamically assess the retinal microvasculature via endothelium-dependent vasodilatory responses [in terms of perfusion and diameter changes, to flicker light (146, 148)].

### Angiographic Techniques

Angiographic methods involve the measurement of transit time of a contrast agent from arteries to veins, which is inversely correlated with blood flow (149, 150). Limitations to this technique are related to diabetes (the sum of all vessel diameters might not be directly related to retinal blood volume) and vasodilation (which alters the contrast distribution volume with an increased circulation time but no changes in blood flow) (149, 150). These measures, made through a scanner laser ophthalmoscopy (SLO) require injection of a contrast agent (151, 152). SLO coupled with adaptive optics (153) and optical coherence tomography angiography (OCT-A) allow for the measurement of all the retinal layers and accurately visualize both retinal and choroidal microvasculature without contrast agent injection (154, 155).

### Laser Doppler Techniques

Laser Doppler techniques are based on the optical Doppler effect, which relies on the reflection of a high coherence laser beam scattered *in vivo* on vascular tissue and captures the shift of the underlying moving red blood cells. The back-scattered light gives a measure of both the incident light (vessel wall) as well as the shifted light (red blood cells), thus providing a measure of relative blood flow, blood volume, and blood velocity within a specified region of the retina. An absolute red blood cell velocity is obtainable by means of bidirectional laser Doppler velocimetry, when the light scattered from the erythrocytes is detected from two directions. For the volumetric blood flow rate calculation, an accurate measure of the diameter is required (156). Laser Doppler flowmetry does not rely on vessel diameter measurement but is based on the intensity of signal derived from the red blood cell volume and velocity (157). Combining the laser Doppler flowmetry with laser scanning tomography, a two-dimensional mapping of retinal blood flow can be obtained, resulting from blood flow measurements based on both single and multiple scattering events from many red blood cells. Local frequency components of the reflected light are obtained at each scanning point and combined with blood velocity (158).

### Other Doppler Techniques

**Figure 4:**
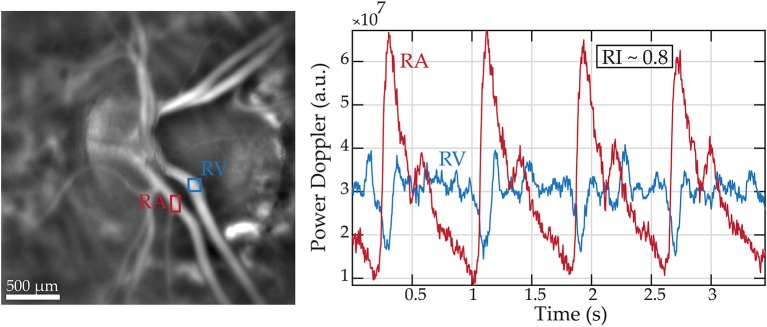
Retinal blood flow measurements in a healthy subject using laser Doppler holography. Left panel: Power Doppler image revealing the vascularized structures. Two regions of interest (ROI) marking a retinal artery and vein are drawn in red and blue, respectively. Right panel: Variations of blood flow over cardiac cycles in the regions of interest.

Combining OCT with the Doppler technique, a simultaneous measure of blood flow and vascular structure and anatomy can be obtained (159). Applied to retrobulbar vessels, color Doppler provides a measurement of PSV and EDV from which RI and pulsatility index can be obtained. Recently, a novel technique has been developed, laser Doppler holography (Figure 4), which overcomes limits of low temporal resolution using previous techniques such OCT-A, allowing a full-field spatio-temporal filtered characterization of retinal small arteries (160).

### Laser Speckle Flowgraphy

Laser speckle flowgraphy is based on an interference phenomenon resulting in a laser speckle pattern changing when a scattered sample moves and allows the measurement of human retinal blood flow in a semi-quantitative fashion. It calculates the pulsatile flow from the difference in the mean blur rate produced by the moving erythrocytes during the systolic and diastolic phase (blowout time and acceleration time index). The blowout time has been inversely associated with age, brachial-ankle PWV and directly correlated with carotid IMT. Studies in healthy subjects observed a correlation between pulsatile flow with carotid artery thickening and high carotid plaque formation (161).

Despite a number of studies examining the relationship between microvascular structural changes at the retinal level and systemic macrovascular disease (162–165), the prognostic value of retinal pulsatility variables remains to be fully elucidated. One recent study showed that impaired retinal microvascular function predicted all-cause mortality in patients with end stage renal disease (148). Given that laser Doppler techniques are the only currently available methods to measure retinal pulsatility, they hold most promise for investigating the interaction between the macro- and microvasculature.

## Summary and Conclusion

Over the last few decades, arterial stiffness has emerged as a major, independent CVD risk factor. There is now ample evidence that arterial stiffening gives rise to increased pressure and flow pulsatility which may be transmitted to the microvasculature and contribute to target organ damage in the brain, kidney, and eye. In this review we have provided a comprehensive summary of the methods to measure the interaction between the macro- and microvasculature. Further understanding the relationship between the macro- and microvasculature and target organs will provide avenues for future treatment and management strategies that can reduce the impact of pulsatility and minimize damage to target organs, lessen the burden of associated disease and ultimately improve survival. Future work should determine whether both lifestyle and pharmacological interventions can regress accelerated arterial stiffening and whether this in turn leads to a reduction in pressure and flow pulsatility and target organ damage.

## Author Contributions

RC and RB contributed conception and design of the study. All authors wrote sections of the manuscript, contributed to manuscript revision, read and approved the submitted version.

### Conflict of Interest

The authors declare that the research was conducted in the absence of any commercial or financial relationships that could be construed as a potential conflict of interest.
